# Phase 2 Open-label, Single-arm, Multi-center Clinical Trial to Evaluate the Efficacy and Safety of Camostat Mesylate in Patients with Protein-losing Enteropathy After Fontan Operation

**DOI:** 10.1007/s00246-025-03859-9

**Published:** 2025-04-14

**Authors:** Woo Young Park, Gi Beom Kim, Sang Yun Lee, Jae Suk Baek, Soo Jin Kim, Jowon Jung, Myung Chul Hyun, Young Tae Lim, HyoungDoo Lee, Hoon Ko, Joowon Lee

**Affiliations:** 1 Department of Pediatrics, Seoul National University Children’s Hospital, Seoul National University College of Medicine, 101, Daehak-Ro, Jongno-Gu, Seoul, Republic of Korea; 2 Department of Pediatrics, Asan Medical Center Children’s Hospital, Ulsan University College of Medicine, Seoul, Republic of Korea; 3 Department of Pediatrics, Sejong General Hospital, Bucheon, Republic of Korea; 4 Department of Pediatrics, Yonsei University College of Medicine, Seoul, Republic of Korea; 5 Department of Pediatrics, School of Medicine, Kyungpook National University, Daegu, Republic of Korea; 6 Department of Pediatrics, Pusan National University College of Medicine, Yangsan, Republic of Korea; 7 Department of Pediatrics, Seoul National University Bundang Hospital, Seongnam, Republic of Korea

**Keywords:** Camostat mesylate, Protein-losing enteropathy, Fontan operation, Stool alpha-1 antitrypsin

## Abstract

Protein-losing enteropathy (PLE) is a serious complication after the Fontan operation with limited treatment options. This phase 2, multi-center, open-label trial evaluated the efficacy and safety of Camostat Mesylate (CM), a serine protease inhibitor, as adjunctive therapy for PLE. Nineteen patients aged 4 years and older with PLE after the Fontan operation were enrolled. CM was administered for six months in addition to their individualized conventional treatments. Assessments were made at 1, 3, and 6 months of CM administration, and at one month after CM discontinuation. Outcomes evaluated were the changes in serum albumin level, stool alpha-1 antitrypsin, and clinical symptoms such as, diarrhea, edema, weight change, and ascites. Of the 19 patients enrolled, 4 voluntarily withdrew consent, and the data from the 15 patients who completed the study were analyzed. Their median age was 15.0 years (interquartile range, 12.0–21.5) and the median time between the Fontan surgery and PLE diagnosis was 2.4 years. Serum albumin levels increased from 2.2 to 2.5 g/dL (*p* = 0.183), while stool alpha-1 antitrypsin levels significantly decreased from 215.6 to 75.5 mg/dL (*p* = 0.016) over six months. Patients with baseline diarrhea showed notable improvements: serum albumin increased from 1.8 to 2.4 g/dL (*p* = 0.138) and stool alpha-1 antitrypsin decreased from 220.3 to 75.5 mg/dL (*p* = 0.075) over 6 months. No serious adverse events occurred. CM demonstrated significant reductions in gastrointestinal protein losses, particularly in patients with baseline diarrhea. Trial registration NCT05474664.

**Supplementary Information:**

The online version contains supplementary material available at 10.1007/s00246-025-03859-9.

## Introduction

The Fontan procedure has allowed patients with functional single ventricle to achieve long-term survival and improved quality of life, with outcomes extending into adulthood [1, 2]. Nevertheless, long-term surveillance of this physiological state has revealed a spectrum of complications [3]. Among these, protein-losing enteropathy (PLE) represents one of the most severe pathological outcomes of the Fontan circulation, associated with increased morbidity and mortality [4]. Although various hypotheses have been proposed, the exact mechanisms leading to PLE development after Fontan operation remain incompletely understood, and the heterogeneity in treatment responses among individuals suggests that there may be multiple underlying potential causes. Furthermore, the low prevalence of PLE limits the literature about its treatment to small observational studies, and there is no single therapy renowned to be effective for all PLE patients [5].

Camostat mesylate (CM), a synthetic serine protease inhibitor that is orally bioavailable, was initially developed and approved for its use in Japan in 1985. It has been clinically used to treat acute exacerbations of chronic pancreatitis and reflux esophagitis within Japan and South Korea [6, 7]. In vitro studies have demonstrated that CM plays a role in managing intestinal conditions by stabilizing epithelial cell adhesion molecules and maintaining epithelial integrity, which is essential for gastrointestinal health [8, 9]. This ability of CM to stabilize epithelial function provides the rationale for using CM in patients with PLE following the Fontan procedure. While no direct research has been conducted to evaluate the CM’s effects on PLE, various animal studies [8, 9] have provided promising evidence that CM could help in resolving intestinal problems by enhancing epithelial stability. Given the complexity of PLE and its severe implications, this research aims to evaluate the potential efficacy and safety of CM in treating PLE after Fontan surgery.

## Method

### Trial Design

This study is a multi-center, open-label, single-arm, phase 2 clinical trial for patients diagnosed with PLE following Fontan operation. Participants were recruited from June 2022 to August 2024. The trial was conducted across six tertiary care hospitals in South Korea. The inclusion criteria were patients aged 4 years and older at the time of consent, who had previously been diagnosed with PLE or were newly diagnosed with PLE after Fontan surgery. PLE diagnosis was confirmed by clinical manifestations such as, ascites, pleural effusion, peripheral edema, diarrhea, or abdominal pain, in conjunction with a serum albumin concentration of 3.0 mg/dL or lower. Objective biomarkers, including stool alpha-1 antitrypsin levels, were also measured to support the diagnosis, although protein-losing scintigraphy was not routinely performed due to clinical limitations. Additionally, patients were required to have no evidence of liver or kidney diseases for at least 3 months prior to enrollment. The exclusion criteria included the patients who could not take the medication orally or had hypersensitivity reactions. Patients with severe dietary restrictions or genetic metabolic diseases, including galactose intolerance and glucose-galactose malabsorption, and those with severe kidney (plasma creatinine > 2.0 mg/dL) or liver diseases (plasma aspartate aminotransferase and/or alanine aminotransferase exceeding three times the normal range), which could negatively affect the absorption, metabolism, or excretion of oral medications were also excluded. The trial was conducted in accordance with Good Clinical Practice and reported in accordance with the CONSORT 2010 statement. The protocol was approved by the Institutional Review Board of the Seoul National University Hospital (IRB number: H-2205-014-1320). Written informed consent was obtained from each participant or from their parents or legal guardians in the case of minors, prior to any trial-related procedures. Trial data were collected by the principal investigators and managed using research electronic data capture (REDCap) hosted at the Clinical Trial Unit of the Seoul National University Hospital.

### Trial Procedures

Study participants were given CM in addition to their current treatment regimen for PLE. To determine the safe pediatric and adult dosages of CM, we referenced dosages utilized in previous studies [10–12]. Children aged between 4 and 13 years were administered 100 mg of CM twice daily, while adolescents and adults aged 13 years and above received 100 mg three times daily. A previous study showed that the median duration from the therapy initiation to the treatment response is 2.5 months [13]. However, it is recognized that the response time to treatment can vary significantly among patients. Therefore, this clinical trial was designed to evaluate the effect of CM therapy over 6 months. The effectiveness and safety of the treatment were evaluated by the trial’s clinical staff at one month, three months, and 6 months after starting CM. Additionally, participants underwent the final evaluation after one month of discontinuing the CM treatment, while maintaining the standard care.

### Outcomes

The primary outcome was the improvement in serum albumin levels compared to baseline. The secondary outcomes included the following: (1) stool alpha-1 antitrypsin concentration at 6 months compared to baseline; (2) improvement in the symptoms of PLE such as the number of daily defecations, presence of diarrhea, edema, changes in body weight, and reduction in ascites volume assessed by ultrasound. Edema was defined as clinically detectable peripheral pitting edema identified on physical examination. The medical dictionary for regulatory activities (MedDRA ver 25.0) grading scale was utilized to classify adverse events. A serious adverse event was defined as any unexpected medical occurrence that, regardless of dosage, leads to death, is life-threatening, requires inpatient hospitalization or prolongation of existing hospitalization, results in persistent or significant disability/incapacity, involves a congenital anomaly/birth defect, or is considered a medically significant event.

### Concomitant Treatment

All patients were receiving medication for PLE treatment. Given that there is no single designated medication for PLE and treatment responses vary among individuals, the PLE medications taken by the participants were diverse. The PLE medications and other related drugs taken by the patients are summarized in Supplementary Table 1. All patients were taking diuretics and anticoagulants including warfarin or aspirin, and about half of the patients were taking angiotensin-converting enzyme inhibitor and phosphodiesterase-5 inhibitor. Whenever albumin replacement therapy was administered within two weeks prior to the blood sampling date, the corresponding albumin values were excluded from the analysis to minimize potential confounding effects.

### Statistical Analysis

The primary analysis was conducted both as intention-to-treat and per-protocol (PP) analysis for the 15 patients who completed the study, excluding those who received < 50% of the planned doses of study medication. Stratified analyses were used to explore potential differences in effect of the intervention among subgroups (± diarrhea at baseline). SPSS version 23.0 (IBM) and GraphPad Prism software were used for data analysis. For the descriptive analysis, continuous variables are described as median with interquartile range, while categorical variables are presented as frequencies. The Wilcoxon signed‐rank test was used to compare variables between baseline and 6-month follow-up, as well as between baseline and the study endpoint. In the subgroup analysis, the Mann–Whitney test was used to compare continuous values, and Fisher’s exact test was used to compare categorical values. Statistical significance was defined as *p* < 0.05.

## Result

### Patients

Between June 14, 2022 and August 31, 2024, a total of 19 participants diagnosed with PLE following the Fontan operation were enrolled. Among these, four patients voluntarily withdrew their consents (2 patients withdrew after the 1-month follow-up and other two after completing the 3-month follow-up) due to a lack of symptom improvement or a perceived worsening of symptoms despite taking the medication. Consequently, a total of 15 patients completed the study. Since the primary outcome focused on the changes in serum albumin levels from baseline to 6 months or to the study endpoint, a PP analysis was conducted. The baseline characteristics of the PP population are summarized in Table 1. Of the 15 participants, 8 (53.3%) were male, and 7 (46.7%) were female. The median age was 15 years, with the youngest being 12 years old. The median body weight was 36.9 kg [interquartile range (IQR), 31.7–44.1], and the median time from Fontan operation to PLE diagnosis was 2.4 years (IQR, 1.0–7.2). At baseline, the median serum albumin level was 2.2 g/dL (IQR, 1.8–3.2), and the median stool alpha-1 antitrypsin level was 215.6 mg/dL (IQR, 96.9–412.0).

**Table 1:** Patients’ baseline characteristics (per-protocol analysis)

Variables	Total *N* = 15
Age, years (median, IQR)	15.0 (12.0–21.5)
Gender (Male/Female)	8/7
Body weight, kg (median, IQR)	36.9 (31.7–44.1)
Time interval from Fontan op. to PLE diagnosis, years (median, IQR)	2.4 (1.0–7.2)
Symptoms	
Stool number (median, IQR)	3.0 (1.5–3.5)
Diarrhea, *N* (%)	7/15 (46.7%)
Edema, *N* (%)	7/15 (46.7%)
Ascites, *N* (%)	10/15 (66.7%)
Laboratory results	
Serum albumin (median, IQR), g/dL	2.2 (1.8–3.2)
Stool alpha-1 antitrypsin (median, IQR), mg/dL	215.6 (96.9–412.0)

*IQR* interquartile range

### Primary Outcome

**Fig. 1:**
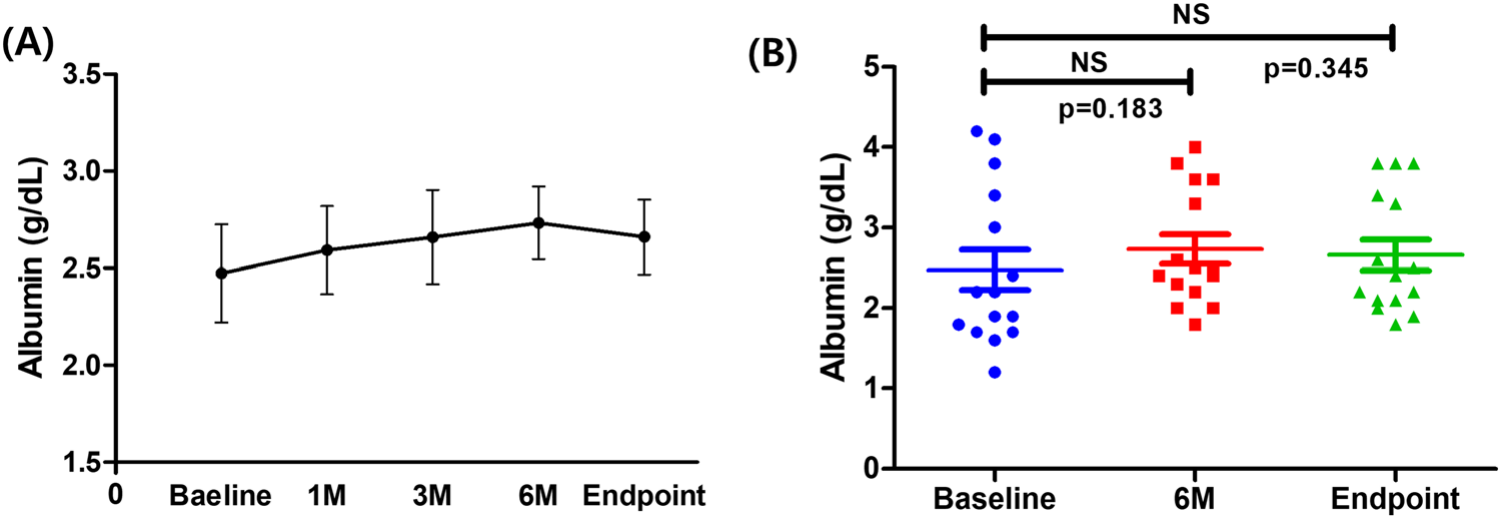
Changes in serum albumin during the study period. **A** Serum albumin levels over the study period, showing an increasing trend until 6 months, followed by a decrease after discontinuation of the medication. **B** Comparison of serum albumin levels at baseline, 6 months, and study endpoint, with no statistically significant differences. (*p* = 0.124, for baseline vs. 6 months, *p* = 0.382, for baseline vs. endpoint)

The primary outcome was to assess the improvement in serum albumin levels at 6 months or at the study endpoint compared to baseline. Median serum albumin increased from 2.2 g/dL (IQR, 1.8–3.2) at baseline to 2.5 g/dL (IQR, 2.3–3.5) at 6 months (*p* = 0.183). Although not statistically significant, this change may indicate a trend toward improvement. At the study endpoint, following treatment discontinuation, a non-significant decrease was observed (2.4 g/dL, IQR, 2.1–3.4; *p* = 0.345) (Fig. 1A, B).

### Secondary Outcomes

**Fig. 2:**
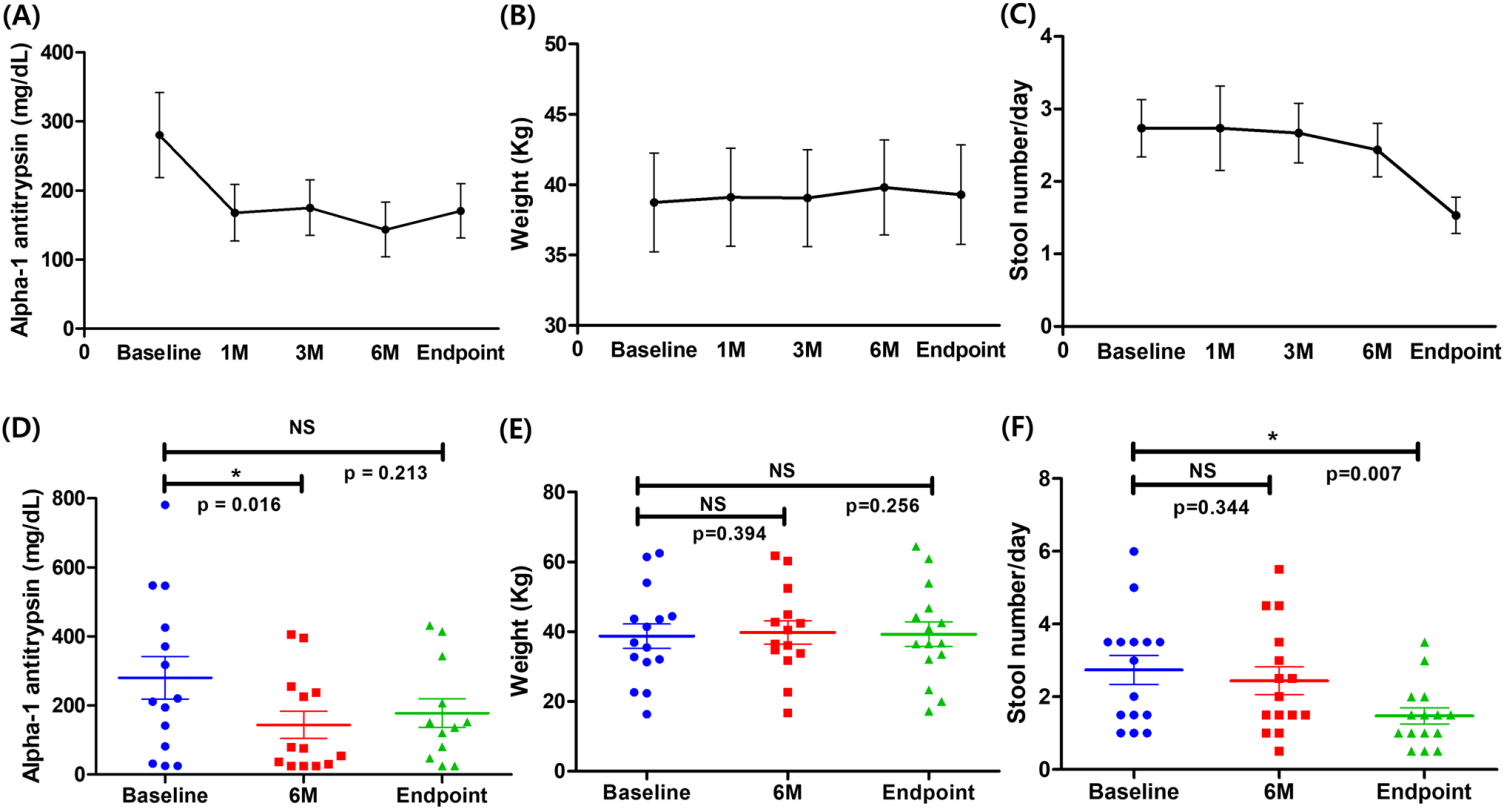
Changes in stool alpha-1 antitrypsin levels, body weight, and stool frequency during the study period. **A** Stool alpha-1 antitrypsin levels over the study period, showing a decreasing trend until 6 months, followed by an increase after discontinuation. **B** Body weight over the study period, showing no statistically significant changes. **C** Daily stool frequency over the study period, showing a significant reduction by the study endpoint. **D** Comparison of stool alpha-1 antitrypsin levels at baseline, 6 months, and study endpoint, with a statistically significant reduction between baseline and 6 months (*p* = 0.016). **E** Comparison of body weight at baseline, 6 months, and study endpoint, with no statistically significant differences. **F** Comparison of daily stool frequency at baseline, 6 months, and study endpoint, with a statistically significant reduction between baseline and study endpoint (*p* = 0.007)

Changes in stool alpha-1 antitrypsin level, body weight, and stool frequency are summarized in Fig. 2. Stool alpha-1 antitrypsin levels significantly decreased from a median of 215.6 mg/dL (IQR, 96.9–412.0) at baseline to 75.5 mg/dL (IQR, 29.6–236.7) at 6 months (*p* = 0.016). At the study endpoint, levels increased numerically to 143.7 mg/dL (IQR, 72.0–241.0), though this change was not statistically significant (*p* = 0.213).

Body weight remained stable throughout the study. Median values were 36.9 kg (IQR, 31.7–44.1) at baseline, 36.5 kg (IQR, 32.8–43.9) at 6 months (*p* = 0.394), and 36.7 kg (IQR, 32.8–45.5) at the study endpoint (*p* = 0.256).

Stool frequency improved significantly from 3.0 times per day (IQR, 1.5–3.5) to 1.5 times per day (IQR, 1.0–1.8) by the study endpoint (*p* = 0.007). Despite these improvements, the grade of ascites assessed by ultrasound and the proportion of patients experiencing diarrhea and edema did not show any statistically significant differences. (Supplementary Fig. 1).

### Subgroup Analysis

A subgroup analysis was conducted to compare the differential responses in serum albumin and stool alpha-1 antitrypsin levels based on the presence of diarrhea at baseline. Patients were divided into two groups: those with diarrhea (*n* = 7) and those without diarrhea (*n* = 8). Baseline characteristics of the two groups are summarized in Table 2. Other than the presence of diarrhea, there were no significant differences between groups in age, sex, body weight, or the time interval from Fontan operation to PLE diagnosis. The diarrhea group had a significantly higher baseline stool frequency (median, 3.5 stools/day) compared with the non-diarrhea group (median, 1.5 stools/day; *p* = 0.009).

**Table 2:** Subgroup analysis according to baseline presence of diarrhea

Variables	Diarrhea group	No Diarrhea group	*p*-value
*N*	7	8	
Age, years	16.0 (14.0 ~ 21.0)	12.5 (10.3 ~ 22.0)	0.463
Gender (M/F)	5/2	3/5	0.315
Baseline body weight, kg	35.5 (32.5 ~ 44.1)	39.2 (29.1 ~ 46.2)	0.779
Time interval OP to PLE diagnosis, years	2.5 (1.2 ~ 8.1)	2.2 (0.6 ~ 7.0)	0.535
Baseline symptoms			
Stool number (median, IQR)	3.5 (3.5 ~ 4.25)	1.5 (1.0 ~ 2.25)	0.009
Edema, *N* (%)	4/7 (57.1%)	3/8 (37.5%)	0.619
Ascites, *N* (%)	5/7 (71.4%)	5/8 (62.5%)	1.000
Laboratory results			
Serum albumin			
Baseline (median, IQR), g/dL	1.8 (1.7 ~ 2.1)	2.7 (2.1 ~ 3.5)	0.076
6 month f/u (median, IQR), g/dL	2.4 (2.3 ~ 3.3)	2.6 (2.5 ~ 3.4)	0.573
Endpoint (median, IQR), g/dL	2.2 (2.0 ~ 3.1)	2.6 (2.2 ~ 3.3)	0.613
Stool alpha-1 antitrypsin			
Baseline (median, IQR), mg/dL	220.3 (176.2 ~ 486.5)	194.1 (56.7 ~ 344.3)	0.559
6 month f/u (median, IQR), mg/dL	75.5 (27.3 ~ 230.7)	66.2 (40.7 ~ 210.9)	0.836
Endpoint (median, IQR), mg/dL	100.3 (38.8 ~ 132.1)	275.0 (165.3 ~ 396.6)	0.041

Values are number or median, Interquartile range

**Fig. 3:**
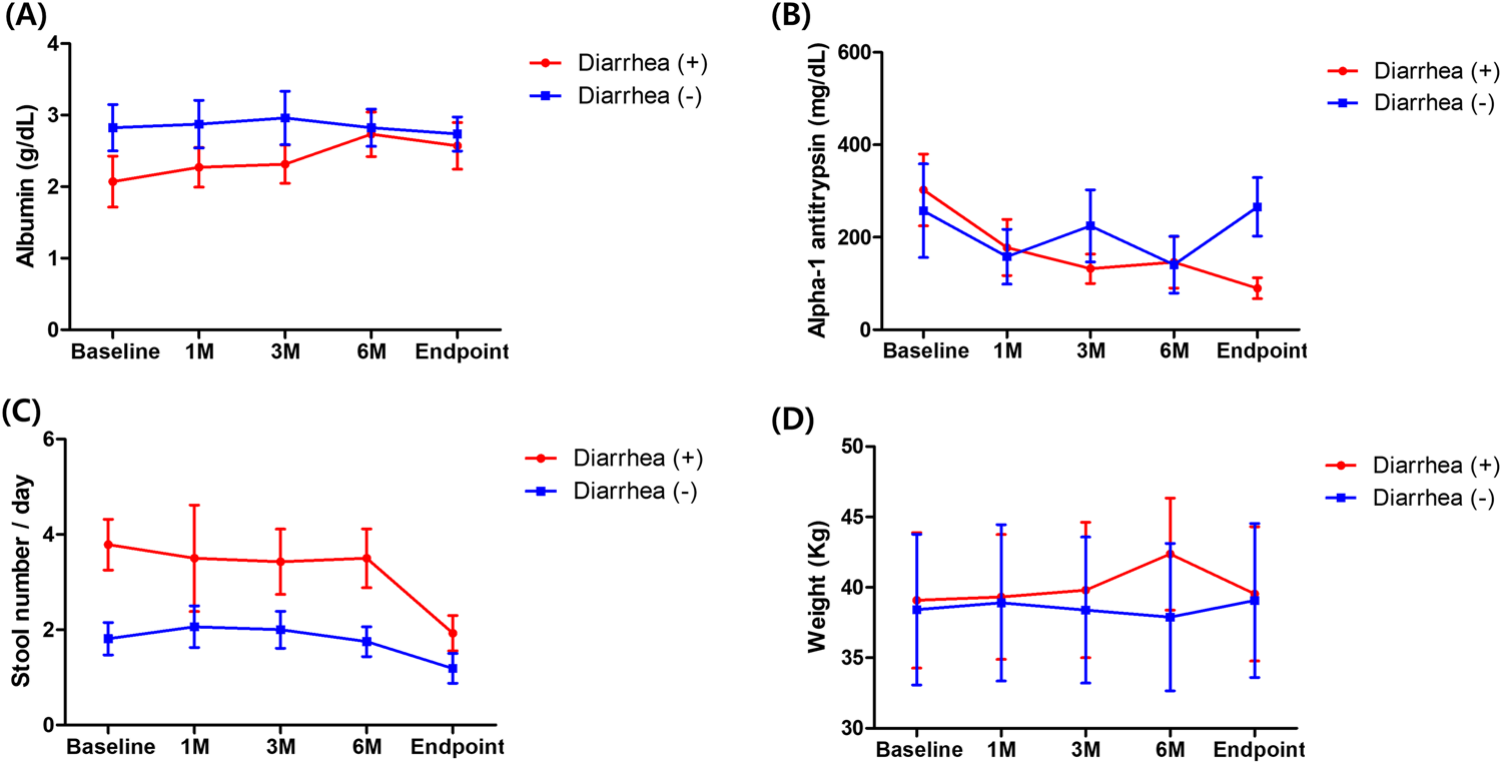
Subgroup analysis of patients based on the presence of diarrhea at baseline. **A** Serum albumin levels over the study period in the two groups. **B** Stool alpha-1 antitrypsin levels in the groups during the study period. **C** Comparison of stool frequency between the groups throughout the study period. **D** Body weight changes in the groups over the study period. Overall, patients with diarrhea demonstrated more favorable treatment responses

Changes in laboratory values and symptoms are shown in Fig. 3. In the diarrhea group, serum albumin increased from 1.8 g/dL (IQR, 1.7–2.1) at baseline to 2.4 g/dL (IQR, 2.3–3.3) at 6 months (*p* = 0.138), while the non-diarrhea group showed a slight decrease from 2.7 g/dL (IQR, 2.1–3.5) at baseline to 2.6 g/dL (IQR, 2.5–3.4) (*p* = 0.623). At the study endpoint, serum albumin levels in the diarrhea group were 2.2 g/dL (IQR, 2.0–3.1) (*p* = 0.116, for baseline vs endpoint), while the non-diarrhea group showed levels of 2.6 g/dL (IQR, 2.2–3.3) (*p* = 0.866, for baseline vs. endpoint).

In the diarrhea group, stool alpha-1 antitrypsin decreased from 220.3 mg/dL (IQR, 176.2–486.5) to 75.5 mg/dL (IQR, 27.3–230.7) at 6 months, with borderline statistical significance (*p* = 0.075). The non-diarrhea group also showed a reduction from 194.1 mg/dL (IQR, 56.7–344.3) to 66.2 mg/dL (IQR, 40.7–210.9) (*p* = 0.116). At the study endpoint, the diarrhea group exhibited a statistically significant decrease to 100.3 mg/dL (IQR, 38.8–132.1; *p* = 0.043), while the non-diarrhea group showed a non-significant increase to 275.0 mg/dL (IQR, 165.3–396.6; *p* = 0.600).

Regarding stool frequency, the diarrhea group showed a significant decrease from a median of 3.5 stools/day (IQR, 3.5–4.3) to 1.5 stools/day (IQR, 1.5–1.8) at the study endpoint (*p* = 0.042). In the non-diarrhea group, stool frequency decreased from 1.5 stools/day (IQR, 1.0–2.3) to 1.0 stools/day (IQR, 0.5–1.25), but the change was not statistically significant (*p* = 0.084). No significant differences were observed in body weight changes in either group over the study period.

### Details of Voluntary Participant Withdrawals

Of the 19 participants enrolled in the study, four withdrew before completing the trial. The reasons for their withdrawal are summarized in Table 3. Two participants experienced mild abdominal discomfort and did not observe an increase in serum albumin levels within the first month of treatment. Based on these observations, they chose not to continue participating in the clinical trial and voluntarily withdrew consent. The remaining two participants, who had pre-existing diarrhea related to PLE, reported persistent diarrhea after three months of treatment. Both patients and their guardians determined that there were no improvements and decided to withdraw consent voluntarily. None of these withdrawals were due to the side effects attributable to CM.

**Table 3:** Characteristics of Participants who withdrew consent from the study

	Reason for withdrawal	Duration of treatment (month)	Baseline/last albumin (g/dL)	Baseline/last alpha-1 antitrypsin (mg/dL)	Withdrawal type	Additional notes
Patient 1	Mild abdominal discomfort, did not feel improvement with treatment	1	3.9/2.4	444.26/NA	Voluntary	No significant adverse effects noted
Patient 2	Mild abdominal discomfort, did not feel improvement with treatment	1	2.0/2.1	383.32/49.1	Voluntary	No significant adverse effects noted
Patient 3	Diarrhea (PLE symptom) persisted despite treatment	3	2.2/1.5	313.1/294.3	Voluntary	No significant adverse effects noted Symptoms related to PLE exacerbation
Patient 4	Diarrhea (PLE symptom) persisted despite treatment	3	2.8/3.0	185.6/338.9	Voluntary	No significant adverse effects noted Symptoms related to PLE exacerbation

### Safety

In this study, adverse events were reported in 8 participants, representing 53.3% of the study cohort. The predominant adverse drug reactions were gastrointestinal symptoms, including abdominal discomfort, diarrhea, and vomiting (4/8, 50%). In addition, 2 participants experienced electrolyte imbalances, 1 experienced worsening of anemia, and 1 reported chest pain. Importantly, no serious adverse events associated with CM were observed during the study. The adverse effects observed were consistent with the established side effect profile of the medication, which had been recognized previously during its approval process. No unexpected adverse events were observed.

## Discussion

This study represents the first investigation of CM’s impact in patients with PLE following the Fontan operation. Despite the limited sample size, the results are encouraging and suggest a potentially effective role for CM in reducing intestinal protein loss, particularly in patients with baseline diarrhea. Stool alpha-1 antitrypsin levels significantly decreased over six months of treatment, and this effect was more pronounced in the subgroup with diarrhea at baseline. Although the attenuation of these effects after treatment discontinuation warrants further investigation, these findings suggest that ongoing CM therapy may be beneficial in sustaining clinical improvement. Larger-scale and long-term studies are needed to confirm these effects of CM in patients with Fontan-associated PLE.

In the subgroup without baseline diarrhea, a reduction in stool alpha-1 antitrypsin levels was observed following treatment; however, this was not accompanied by an increase in serum albumin concentrations. This apparent dissociation suggests that serum albumin levels in this population may be influenced by factors beyond gastrointestinal protein loss. Possible explanations include impaired hepatic albumin synthesis, persistent systemic inflammation, or compromised nutritional status, all of which may contribute to hypoalbuminemia independently. Additionally, the absence of diarrhea may reflect a less severe or differently manifested intestinal phenotype, in which the therapeutic effect of CM on enteric protein loss does not sufficiently impact systemic albumin homeostasis. These findings highlight the complexity of albumin regulation in patients with Fontan-associated PLE and underscore the need for further investigation into the interplay between intestinal protein leakage and systemic protein metabolism.

To better understand these subgroup-specific findings, it is essential to consider the broader pathophysiologic mechanisms underlying PLE in the Fontan population. The pathophysiology of PLE following the Fontan operation remains unclear; however, a proposed mechanism involves a combination of various factors. The Fontan circulation depends on increased systemic venous pressure (SVP), with early postoperative SVP > 12 mmHg linked to a higher risk of PLE [14]. Additional factors that elevate SVP, including Fontan pathway obstruction, aortic valve regurgitation, and pulmonary artery branch stenosis, are implicated in up to 80% of PLE cases [15], and could represent reversible causes. However, since only a subset of patients with elevated SVP develops PLE, the condition’s pathogenesis is likely multifactorial. Contributing factors may include chronic low cardiac output, which reduces intestinal perfusion and promotes a proinflammatory state, along with altered enterocyte membrane permeability [16]. Abnormal lymphatic circulation, such as lymphatic congestion and lymphangiectasia, has also emerged as a significant contributor to protein leakage [17–19]. Furthermore, variability in lymphatic anatomy, as well as factors like viral infections [20] or genetic predisposition [21], may trigger PLE even in patients with optimal Fontan hemodynamics.

In this study, we focused on the changes in the intestinal environment in PLE patients caused by various mechanisms. Our hypothesis was that an exogenous serine protease inhibitor, known to preserve intestinal epithelial architecture and shown to be effective in treating intestinal failure in vitro [8, 9], could have a beneficial impact on the management of PLE following Fontan surgery. Patients with PLE after Fontan surgery are known to have higher levels of hepatocyte growth factor (HGF) compared to those without PLE, though the clinical significance remains unclear [22]. HGF, which binds to the MET receptor and promotes cell growth and regeneration, is activated by hepatocyte growth factor activator and cellular type II transmembrane serine proteases. These proteases are regulated by hepatocyte growth factor activator inhibitors (HAIs), specifically HAI-1 and HAI-2, which are crucial for maintaining intestinal mucosal integrity [9]. HAI-1 and HAI-2 function to prevent excessive or inappropriate activation of HGF, and we hypothesize that CM potentially supports these regulatory mechanisms, thereby contributing to the preservation of intestinal epithelial integrity. Furthermore, it is hypothesized that among Fontan PLE participants, those with baseline diarrhea may have had more severely damaged intestinal epithelial barriers, explaining the more pronounced effect of CM observed in this subgroup. However, further studies are needed to elucidate the detailed mechanisms involved.

Due to the low prevalence of PLE, the literature on treatment is limited to small observational studies, with treatment responses being highly heterogeneous and difficult to predict. No single treatment is universally effective, and most therapies for PLE may take several months to show measurable outcomes. Therefore, therapy should be individualized, focusing on minimizing symptoms, reducing side effects, and improving quality of life. In this study, CM was administered in addition to each patient’s current PLE medications, which are summarized in Supplementary Table 1. Given the variability in treatment responses, the medication was administered for six months, and to assess any changes after discontinuation, the last follow-up was conducted one month after stopping the treatment. The results showed that stool alpha-1 antitrypsin levels improved over the six-month period, and suggested an improvement in serum albumin level. Both parameters worsened after drug discontinuation, indicating that ongoing therapy might be required to maintain the treatment effects. This finding suggests the need for the further research to determine the optimal duration of treatment.

Our results suggest that Fontan patients did not experience any additional side effects related to CM beyond those that were previously reported. Among the 15 participants, adverse events were reported in 8 individuals. Importantly, there were no serious adverse events, no adverse events leading to treatment discontinuation, and no adverse events or adverse drug reactions leading to death.

### Limitations

This pilot trial had several limitations. First, since Fontan PLE is a rare disease, the small sample size makes it challenging to achieve statistical significance and may lead to reduced statistical power. Nonetheless, considering the rarity of the condition, the inclusion of 15 patients in this study is noteworthy. To address the limitation of small sample size, a multinational, multi-center study may be necessary. Second, the follow-up duration was relatively short, limiting our ability to determine the optimal duration of medication or to assess long-term changes after discontinuation. Therefore, future studies with extended long-term follow-up are needed.

## Conclusion

In conclusion, this study suggests that CM may be a potentially effective and tolerable treatment for PLE following the Fontan operation, especially in patients with baseline diarrhea.

## Supplementary Information

Below is the link to the electronic supplementary material.

Supplementary file1 (TIF 720 KB)—Symptom proportions and ascites grade over the study period proportions of patients reporting (A) diarrhea and (B) edema during the study period, and (C) comparison of ascites grade assessed by sonography. Although there is a trend toward symptom improvement after 6 months, no statistically significant changes were observed in sonographically assessed ascites grade

Supplementary file2 (DOCX 15 KB)

## Data Availability

The data that support the findings of this study are available from the corresponding author upon reasonable request.

## Abbreviations

CM: Camostat Mesylate
HAIs: Hepatocyte growth factor activator inhibitors
HGF: Hepatocyte growth factor
PLE: Protein-losing enteropathy
PP: Per-protocol
SVP: Systemic venous pressure
