# Supplementary figures and images for: Phase 2 Open-label, Single-arm, Multi-center Clinical Trial to Evaluate the Efficacy and Safety of Camostat Mesylate in Patients with Protein-losing Enteropathy After Fontan Operation
Source: Pediatr Cardiol. 2025 Apr 14;47(3):932–40. doi: 10.1007/s00246-025-03859-9 (PMC12901255; doi:10.1007/s00246-025-03859-9)

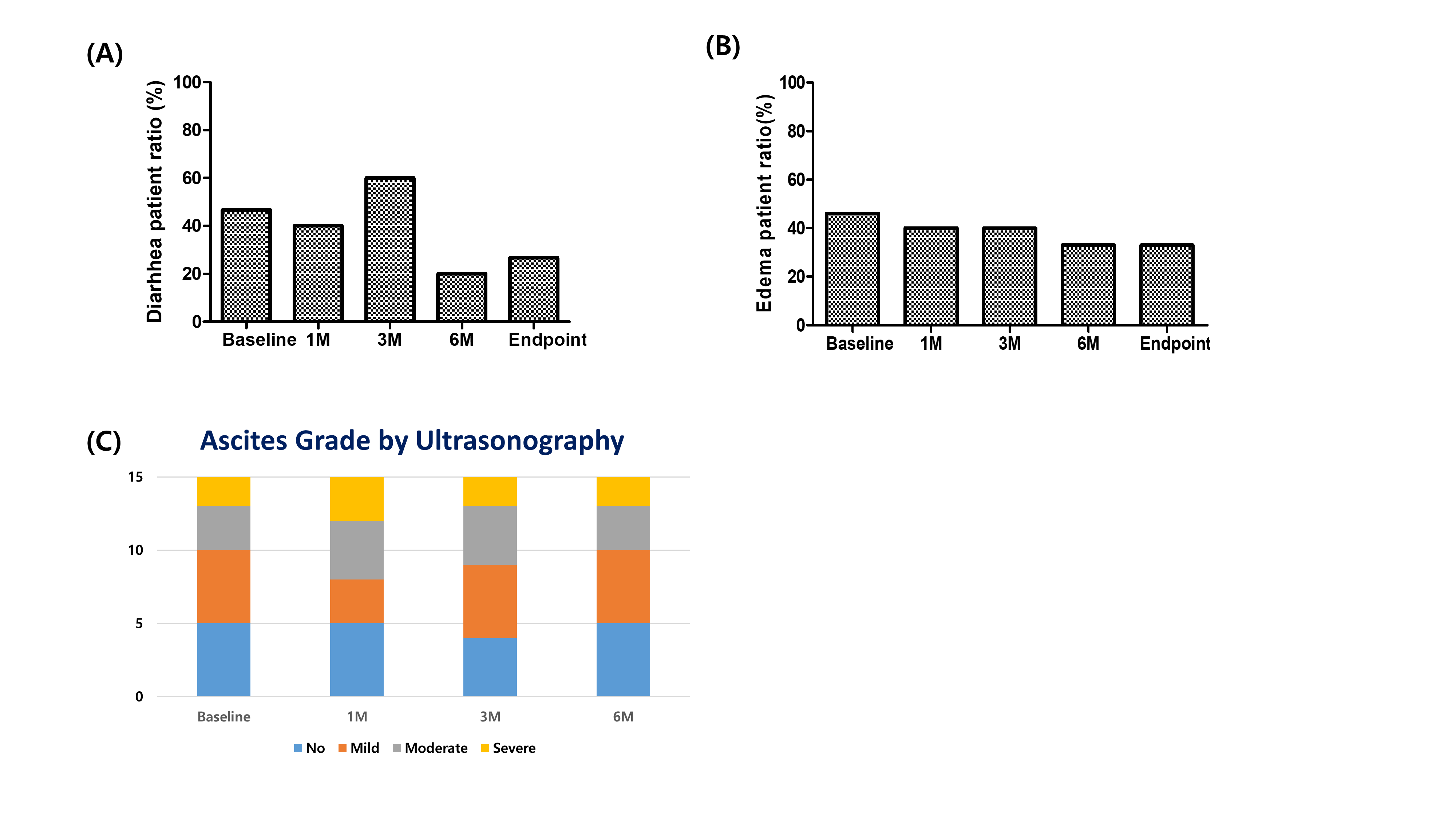

Supplement: Supplementary file 1 — Supplementary file1 (TIF 720 KB)—Symptom proportions and ascites grade over the study period proportions of patients reporting (A) diarrhea and (B) edema during the study period, and (C) comparison of ascites grade assessed by sonography. Although there is a trend toward symptom improvement after 6 months, no statistically significant changes were observed in sonographically assessed ascites grade [file 246_2025_3859_MOESM1_ESM.tif]
