# Supplementary material for: Phase 2 Open-label, Single-arm, Multi-center Clinical Trial to Evaluate the Efficacy and Safety of Camostat Mesylate in Patients with Protein-losing Enteropathy After Fontan Operation
Source: Pediatr Cardiol. 2025 Apr 14;47(3):932–40. doi: 10.1007/s00246-025-03859-9 (PMC12901255; doi:10.1007/s00246-025-03859-9)
Supplement: Supplementary file 2 — Supplementary file2 (DOCX 15 KB) [file 246_2025_3859_MOESM2_ESM.docx]

| Number | Furosemide | Spironolactone | Thiazide | LMWH | Steroid | Immunoglobulin | ACE-I | PDE5-I | ERAs | Albumin | Probiotics | Warfarin | Aspirin |
| --- | --- | --- | --- | --- | --- | --- | --- | --- | --- | --- | --- | --- | --- |
| 1 | O | O |  |  | O |  | O |  |  | O |  | O |  |
| 2 | O | O |  | O |  |  |  |  |  | O | O |  | O |
| 3 | O | O |  |  |  |  |  | O |  | O | O | O |  |
| 4 | O | O |  |  |  |  | O | O | O |  |  |  |  |
| 5 | O | O | O |  | O |  |  | O |  |  |  | O |  |
| 6 |  | O |  |  | O |  | O |  |  |  | O | O |  |
| 7 | O | O |  |  |  |  |  |  |  | O |  | O |  |
| 8 | O | O |  | O |  |  |  |  |  | O |  |  | O |
| 9 | O |  |  |  |  |  | O | O |  |  |  | O |  |
| 10 |  | O |  |  |  |  | O |  |  |  |  |  | O |
| 11 | O | O |  | O |  | O |  |  |  | O | O |  | O |
| 12 | O | O |  |  | O |  | O | O |  |  |  | O |  |
| 13 | O | O |  | O |  |  | O | O |  |  |  |  | O |
| 14 | O | O |  |  |  |  | O | O |  |  |  | O |  |
| 15 |  | O |  |  |  |  |  | O |  |  | O | O |  |

Supplementary table 1. Concomitant PLE-Related Medications Taken by Participants

Note: LMWH, low molecular weight heparin; ACE-I, angiotensin-converting enzyme inhibitor; PDE5-I, Phosphodiesterase Type 5 Inhibitor; ERAs, Endothelin Receptor Antagonists
